# Annual change in estimated glomerular filtration rate and predictors of rapid kidney function decline among antiretroviral therapy-experienced older people with HIV in Uganda, sub-Saharan Africa: A cohort study

**DOI:** 10.21203/rs.3.rs-10903834/v1

**Published:** 2026-11-01

**Authors:** Amutuhaire Judith Ssemasaazi, Rebecca Scherzer, Peter Messiaen, Robert Kalyesubula, Barbara Castelnuovo, Michael Shlipak, Michelle Estrella

**Affiliations:** Infectious Diseases Institute; Kidney Health Research Collaborative; Hasselt University; Makerere University; Infectious Diseases Institute; Kidney Health Research Collaborative; Kidney Health Research Collaborative

**Keywords:** Older people, HIV, Chronic kidney disease, Rapid kidney function decline, Tenofovir disoproxil fumarate, kidney function monitoring

## Abstract

**Introduction:**

: Older people with HIV (PWH) receiving antiretroviral therapy (ART) in sub-Saharan Africa are at high risk of chronic kidney disease (CKD) while routine kidney function monitoring remains limited in this setting. We examined changes in estimated glomerular filtration rate (eGFR) per year and risk of rapid kidney function decline over 3 years among older PWH in Uganda.

**Methods:**

Our cohort study included 500 PWH aged ≥ 60 years receiving ART at the Infectious Diseases Institute in Uganda who were followed between 2020–2024. We measured serum creatinine at each annual visit. We estimated predictors of annual eGFR change and rapid kidney function decline (defined as eGFR loss ≥5mL/min/1.73m^2^/year) in linear mixed effects and modified Poisson regression models, respectively.

**Results:**

Among 500 PWH with a median age of 64 (interquartile range (IQR): 62,68) years, 49% were female, and their median eGFR was 75 (IQR: 60,92) mL/min/1.73m^2^ at baseline. Over a median follow up of 2.95 years, the cohort experienced a −0.7 (IQR: −3.3,4.7) mL/min/1.73m^2^ change in eGFR per year, and 76 (15.2%) participants developed rapid kidney function decline. Female sex (1.82 [95%CI:0.95–2.70]), use of integrase strand transfer inhibitors (2.50[95%CI:1.52–3.48]), higher CD4 cell count (0.21[95%CI:0.04–0.37]), and hepatitis B surface antigen positivity (4.19[95%CI:2.11–6.27]) at baseline were associated with improvement in eGFR (mL/min/1.73m^2^) per year. Longer cumulative exposure to tenofovir disoproxil fumarate (TDF) (relative risk (RR) = 1.33 (95%CI:1.11–1.60) per doubling of TDF exposure in years), and male sex (RR = 1.91 (95%CI:1.08–3.37)) were associated with a higher risk of rapid kidney function decline. A higher CD4 cell count (RR = 0.91 per 100 cells increase/ml; 95% CI: 0.84–0.99)) and use of integrase strand transfer inhibitors (RR = 0.41; 95%CI: 0.23–0.72) were associated with a reduced risk of rapid kidney function decline. Switching from TDF prior to or during the cohort did not mitigate the risk of rapid kidney function decline.

**Conclusions:**

Among older PWH in sub-Saharan Africa, annual eGFR decline was mild overall and comparable to other aging populations. However, a subset of this population experienced rapid kidney function decline especially if they were males and those exposed to TDF, and these may need tailored or more frequent kidney function monitoring.

## Introduction

Chronic kidney disease (CKD) is a major public health problem globally (1) and is expected to become the 5th leading cause of death worldwide by 2040 (2). More than half of adults affected by CKD reside in low income countries (LIC), including in sub-Saharan Africa (SSA), often with limited access to routine kidney function monitoring and life-saving kidney replacement therapies (3, 4). Globally, diabetes, hypertension and aging remain the most important traditional risk factors for CKD (5), but HIV has emerged as an independent major risk factor (6–8). Several underlying mechanisms can explain this, including direct HIV effects on the kidneys, exposure to some potentially nephrotoxic antiretroviral therapies (ART) such as tenofovir disoproxil fumarate (TDF) and chronic systemic inflammation (9–15). Additionally, the success of ART has made aging with HIV a reality (16).

As documented by the United Nations Programme on HIV/AIDS (UNAIDS), ≥ 25% of the global population of people with HIV (PWH) are aged ≥ 50 years (17). Sub-Saharan Africa hosts ≥ 60% of the global population with HIV and there is a growing demography of older PWH (17). Within SSA, older PWH represent a high-risk group whose kidneys may accumulate kidney damage through the confluence of infectious and non-infectious causes, such as diabetes and hypertension and age-related nephrosclerosis (6, 10, 18).

Yet, estimated glomerular filtration rate (eGFR) trajectories and CKD progression among PWH remain poorly studied in SSA and other LIC settings where frameworks are not well established for screening and longitudinal monitoring of kidney function (19–21). This lack of guidance, along with limited access to healthcare, leads to delays in the diagnosis and treatment of CKD (22). To inform efforts toward more consistent kidney disease monitoring, we capitalized on an existing cohort in Uganda to examine the rate and predictors of annual eGFR change and rapid kidney function decline (RKFD) among ART experienced PWH aged ≥ 60 years.

## Methods

### Study design

This was a cohort study among PWH aged at least 60 years.

### Study setting and population

Between 2020 and 2024, the “Diagnosis and treatment of non-communicable diseases and geriatric syndromes among older people with HIV in SSA” cohort enrolled and followed 500 PWH aged ≥ 60 years receiving ART at the Infectious Diseases Institute (IDI) Clinic at Mulago, Kampala. The IDI, an institute of Makerere University College of Health Sciences, is a center of excellence for HIV and related infectious diseases care and research. The main clinic at Mulago has ≥ 8000 PWH receiving ART in active follow-up and ≥ 2000 of them are aged ≥ 60 years. Between 2020 and 2021, we consecutively enrolled 500 PWH aged ≥ 60, who provided informed consent to participate and comply with all the scheduled study visits and procedures. Participants were excluded if they were participating in an ongoing clinical trial at the time. Each participant had four annual study visits. Laboratory measurements were done in the IDI core laboratory and Uganda’s Ministry of health Central public health reference laboratory, both internationally accredited laboratories.

### Data collection procedures and study variables

We collected data into an electronic questionnaire using Research Electronic Data Capture (REDCap) (23). We collected data on sociodemographic characteristics (age, sex, gender), comorbidities (diabetes, hypertension, heart failure, stroke) and history of smoking and alcohol use. HIV and related treatment data (ART regimens, years of ART use, history of TDF use or switching from TDF, time of TDF exposure in years) were collected. TDF switching was further classified into having switched from TDF prior to cohort enrollment (“prior switch” past TDF use or prevalent TDF switch) or having switched off of TDF during the follow-up time. Current TDF users were those who were on TDF at cohort enrollment and continued using it throughout their time in the cohort, while participants were classified as “never used” if they had never been on a TDF-based regimen. At baseline, data on their history of co-infections (tuberculosis and active hepatitis B virus [HBV] based on a positive HBV surface antigen test (HBsAg)) were extracted from the electronic clinic database routinely used for patients’ data at IDI. We also collected information on use of non-steroidal anti-inflammatory drugs (NSAIDs). We measured blood pressure using an automated blood pressure machine, with the participant seated upright and an average of three recordings was registered as the reading at each visit. We considered hypertension as a systolic blood pressure ≥ 130 mm Hg or diastolic blood pressure ≥ 80 mm Hg or use of blood pressure lowering medication for those who had a prior diagnosis. Each participant had a random blood glucose (RBG) measured using a point-of-care on-call glucometer. Participants with symptoms of diabetes and a raised RBG (> 11.1mmol/l) were considered diabetic. Otherwise, participants without symptoms but a raised RBG were retested and considered diabetic if their fasting blood glucose (FBG) was >7mmol/l. We defined diabetes as either: i) the use of diabetes medications ii) FBG >7mmol/l without symptoms of diabetes; or iii) RBG > 11.1mmol/l with symptoms of diabetes. We also measured height in meters and weight in kg for each participant using standardized height and weight scales and estimated the body mass index (BMI) in kg/m^2^. Each participant provided blood samples for measurement of CD4 T-lymphocytes, HIV RNA viral load, serum creatinine and urine for dipstick protein detection at each visit. We classified the HIV viral load as detectable if ≥ 50 HIV RNA copies/ml were found. Serum creatinine was measured using an isotope dilution mass spectrometry traceable enzymatic-assay on Roche’s COBAS INTERGRA chemistry analyzer platform while urine protein was measured using urine dipsticks. We calculated the eGFR using the 2021 creatinine-based CKD-EPI Eq. (24). We defined CKD as an eGFR ≤ 60 mL/min/1.73m^2^ and RKFD as an eGFR decline of ≥5ml/min/1.73m^2^ /year, as per KDIGO guidelines (25, 26). We selected this definition which is recommended by KDIGO and has been shown to identify those at high risk of progression to kidney failure, cardiovascular disease (CVD) and mortality (25–27). On dipstick urinalysis, urine protein was graded as negative, trace (< 30mg/g), + 1(30mg/g), ++(100mg/g) or ≥+++ (300mg/g). Participants were assigned as having proteinuria if the dipstick detected ≥ 30mg/g (+ 1) protein.

### Data analysis

We summarized categorical variables as frequencies and percentages, and continuous variables as medians with interquartile ranges (IQR). We used chi-square or Fishers Exact test and Wilcoxon rank-sum test for comparisons of distribution of categorical and continuous variables, respectively. We log-transformed continuous variables with skewed distributions (such as years on TDF) in our regression models.

We calculated the annual change in eGFR using the differences in eGFR between consecutive years and averaged these differences to obtain a participant-specific mean annual eGFR change. To understand predictors of longitudinal eGFR change, we used Bayesian model averaging (BMA) (28) to identify a parsimonious set of risk factors that was independently associated with change in eGFR, retaining predictors with posterior probabilities > 30%. This method accounts for model uncertainty in variable selection by averaging over all candidate models, each weighted by its posterior model probability. Variables selected by BMA were subsequently included in a linear mixed effects model with random intercepts and slopes and an unstructured covariance structure to account for within-person correlation across repeated eGFR measurements. Risk factor by time interaction terms were included to assess differences in eGFR slope across each risk factor. We further performed sensitivity analyses including a selected set of predictors in the mixed effects linear regression model based on information from previous literature that associates those predictors with eGFR change. We observed lower baseline eGFR among participants who died or were lost to follow-up, raising the possibility that informative censoring could bias the estimated eGFR trajectory toward an artificially favorable pattern. We therefore adjusted estimates using inverse probability of censoring weighted (IPCW) analysis to account for potential bias due to loss to follow-up (29). Specifically, we modeled each participant’s probability of being censored based on the list of covariates (age, gender, baseline eGFR, clinical characteristics, and HIV-related factors). Weights were stabilized and applied to the longitudinal model of eGFR. We assessed covariate balance using absolute standardized mean differences before and after weighting, with values below 0.10 considered indicative of adequate balance. We examined the distribution of the stabilized weights and compared baseline eGFR between the complete and censored samples. This plot showed substantial overlap in the baseline eGFR distribution between those who completed the study and those who dropped out, meaning that the positivity assumption is not violated.

We modelled baseline predictors of RKFD in multivariable modified Poisson models with robust standard errors. All candidate predictors were included in this multivariable model, and variables with a p-value < 0.05 were considered significant. To account for the baseline differences in eGFR among our participants, we further performed a sensitivity analysis in which we adjusted our modified Poisson model for baseline eGFR. We used variance inflation factors to evaluate multicollinearity between covariates.

Bayesian model averaging was performed using the BMA package from the R statistical computing language (R Development Core Team, Vienna, Austria). All other analyses were conducted using the SAS system, version 9.4 (SAS Institute, Inc., Cary, NC) and Stata Statistical Software: Release 19. College Station, TX: StataCorp LLC.

## Results

### Characteristics of participants

Among the cohort of 500 PWH aged ≥ 60 years, the median age was 64 (IQR:62–68) years, 49% were female, and median ART duration was 14 (IQR: 9–16) years at baseline (Table 1). Only 8% (n = 40) had a plasma HIV viral load ≥ 50 copies/ml. Overall, 96% (n = 481) were past or current users of a TDF-based ART regimen but only 79% (n = 379) of these were on a TDF-based regimen at the time of enrollment, while 14% (n = 66) had been switched from TDF prior cohort enrollment.

At baseline, median eGFR was 75 (IQR: 60,92) ml/min/1.73 m2 overall, and 25% of the participants had CKD. Females had a significantly lower median eGFR (70 vs. 80 mL/min/1.73m^2^) and a higher prevalence of CKD (33% vs. 18%) than males at baseline, although there was no sex difference in the prevalence of proteinuria.

During follow-up, the proportion of individuals with CKD increased, with the prevalence of CKD increasing steadily among males (Fig. 1)

### Predictors of changes in longitudinal eGFR

During the median follow-up time of 2.95 (IQR: 2.88–3.08) years, 28 (6%) participants died and 50 (10%) were lost to follow-up. Compared to participants who remained in the study, participants who died or lost to follow-up had a significantly lower eGFR and a higher prevalence of CKD at baseline.

During follow-up, the median annual change for the was − 0.7 (IQR: −3.3, 4.7) mL/min/1.73m^2^. Among participants who completed follow-up, 236 (55.9%) experienced a loss in eGFR from baseline, among whom the median annual eGFR change was − 3.0 (IQR: −5.7, −1.3) ml/min/1.73m^2^, Fig. 2.

Of the potential variables, only female sex, HBsAg seropositivity and use of integrase strand transfer inhibitors (INSTIs) had posterior probabilities ≥ 30% and were selected for inclusion in the mixed effects linear regression model. Female sex (n = 243), positive HBsAg (n = 21), and use of INSTIs (n = 327), at baseline were associated with relative improvements in the annual eGFR, with slopes of 1.82 (95%CI:0.95–2.70), 4.19 (95%CI:2.11–6.27) and 2.50 (95%CI:1.52–3.48) mL/min/1.73 m^2^/year, respectively, (Table 2). In a sensitivity analysis, we additionally adjusted for age, systolic and diastolic blood pressure, diabetes, proteinuria, HIV viral load, prior diagnosis of tuberculosis and CD4 cell count in the model based on clinical significance and found that higher CD4 count (per 100 cells/ml) at baseline was associated with a 0.21 (0.04, 0.37) mL/min/1.73m^2^ increase in eGFR per year. (**Supplementary Table S1**). The sensitivity analysis performed after inverse probability weighting showed similar results (**Supplementary Table S2**).

### Risk factors for rapid kidney function decline among older PLWH

A total of 76 (15.2%) participants experienced RKFD. Their median annual change was − 7.2 (−9.7, −5.3) mL/min/1.73m^2^, Fig. 2. Among them were more males, 50 (66%) than females, 26(34%), p-value = 0.01 and 73 (96%) had ever been on a TDF based regimen. However, compared to the rest of the cohort, participants who experienced RKFD had a higher eGFR at baseline, 89 (73–96) vs. 73 (59–89), p-value < 0.01.

In the multivariable modified Poisson regression model, greater exposure to TDF (log_2_TDFyears) and male sex were associated with higher risks of RKFD, with relative risk (RR) estimates of 1.33 (95%CI:1.11–1.60) and 1.91 (95%CI:1.08–3.37), respectively. In contrast, higher baseline CD4 cell count was associated with a lower risk of RKFD (RR = 0.91 per 100 cells/ml; 95% CI: 0.84–0.99), as was use of an INSTIs-based regimen at baseline (RR = 0.41; 95%CI: 0.23–0.72) (Table 3).

In a sensitivity analysis, where we adjusted the Poisson model for baseline eGFR, the association of TDF exposure strengthened to 1.47 (95% CI: 1.21–1.78) while switching off of TDF during the cohort (n = 121) did not appear to reduce the risk of RKFD, but rather was associated with higher risk (RR = 1.88; 95% CI: 1.09–3.24). Also, prior use of TDF (switch from TDF) before cohort enrollment (n = 66) appeared to be associated with higher risk of RKFD (RR = 2.36; 95% CI: 1.00–5.59). Both higher baseline CD4 cell count and use of INSTIs at baseline remained associated with lower risk of RKFD (**Supplementary Table S3**).

## Discussion

In this cohort of older PWH in SSA, we found that the prevalence of CKD increased during follow-up. Although the cohort change in eGFR annually was not larger than the age-expected rate,(30, 31) a sub-group of approximately 1 in 6 older PWH rapidly lost eGFR. We also found that despite the protective effect offered by immune recovery and the consistent renal safety of dolutegravir, prolonged exposure to TDF increased the risk of RKFD and stopping TDF did not appear to restore kidney function in this older population. We also found sex differences in both the prevalence of CKD and loss of eGFR: females had higher prevalence of CKD at any time in the cohort, but males lost significantly more eGFR than females. These findings imply that reliance on population average estimates for eGFR change may overlook the subgroups that are at high risk of rapidly losing kidney function, and this may mask the need for proactive tailored interventions for such high-risk groups. Though studies have not particularly explored eGFR change in older PWH aged ≥ 60, our findings are consistent with those in other populations of PWH which have shown that rapid eGFR decline occurs commonly (32–34) and that older age is associated with more rapid eGFR decline (34–36). Previously, in the general population and among PWH, CKD risk is known to increase with age (37–40). Age-related nephrosclerosis causes eGFR to decline expectedly; however, in PWH, this phenomenon may be exacerbated by the effects of HIV, potentially nephrotoxic ART, chronic systemic inflammation, and multiple comorbidities (10, 40).

Higher CD4 cell counts consistently protected kidney function in this study. These findings align with those from other studies (41, 42) and complement the narrative that use of effective ART treatments prevents non-AIDS related morbidity and mortality (43, 44). Additionally, INSTIs, particularly dolutegravir which was used by 65% of participants in this cohort, were protective against kidney function loss. Dolutegravir affects creatinine secretion without necessarily causing kidney injury which may appear to lower eGFR in the first few days to weeks on the drug, but eGFR usually stabilizes (45–47).

Our findings also highlight the deleterious effects of cumulative TDF exposure on kidney function among older PWH in SSA. In this cohort, the risk of RKFD increased by ≥ 30% per two-fold higher years of TDF exposure. The median exposure to TDF was 5 (IQR: 3.7–8.9) years, so the highest quartile of exposure (median 8.9 years) had approximately 1.43% (95%CI: 1.12–1.85) risk of RKFD compared with the lowest quartile (median 3.7 years). Elsewhere, cumulative TDF exposure has been associated with eGFR decline (48–50). However, unlike prior studies, in our study of older PWH, TDF cessation did not result in kidney function improvement or even normalization of risk. Older PWH may have limited capacity to recover kidney function (40, 51) following insults such as TDF proximal tubular injury. In addition, TDF continues to be the first choice for ART in Uganda and SSA, unless eGFR is < 60 mL/min/1.73m^2^ (52). However, there is also limited kidney function monitoring in routine care (19), thus TDF switching may happen when the eGFR loss is already too substantial to ensure optimal recovery. In studies elsewhere, early switching from TDF, defined by eGFR > 60 mL/min/1.73m^2^ at the time of discontinuation, increased the likelihood of complete eGFR recovery (50, 53, 54). However, even in high-resource settings with more accessible kidney function monitoring, more than one-third of patients who discontinue TDF did not achieve full kidney function recovery (53, 55), and older age (55) and longer TDF exposure (56) were risk factors for incomplete kidney function recovery. Our paper extends these findings to older PWH in SSA.

We further found that male sex was associated with faster annual kidney function decline and increased risk of RKFD compared with females. Although male participants had higher prevalence of hypertension and diabetes, both traditional risk factors for eGFR loss (5, 57), the observed sex differences persisted after multivariable adjustment. These findings are consistent with prior studies in the general population (58, 59). Some studies have demonstrated the role of testosterone in eGFR loss (60, 61). Literature from studies in people receiving gender affirming hormones, show that compared to estrogen, testosterone therapy is associated with reduced renal plasma flow, reduced measured GFR, raised creatinine (62, 63) as well as increase in kidney tubular injury markers (60). Literature further suggests that testosterone may promote apoptosis, inflammation and fibrosis in the kidneys (64, 65). Further research may examine the role of biological sex in the progression of CKD including in the context of treated HIV in SSA.

Paradoxically, HBsAg positivity was associated with improvements in eGFR over the follow-up period in our study, although HBV is known to cause membranous or membrano-proliferative nephropathy and previous studies have mostly pointed to HBV as a risk for development of CKD (66). Given the small proportion (4%) of participants with HBV in our study, our findings need to be examined in a larger cohort with a greater proportion of HBsAg-positive individuals.

We did not observe an association between traditional CKD risk factors as diabetes and hypertension or smoking with eGFR change or RKFD (5). This could partly be due to the fact that these are participants who are in contact with healthcare and their diabetes and hypertension may be largely well controlled. Similarly, we did not observe an association between proteinuria and eGFR loss. Our cohort were largely non-proteinuric as only 72 participants had ≥ 1 + dipstick proteinuria at baseline in this cohort. However, we also relied on dipstick proteinuria which is less sensitive than urine albumin quantification, and may have led to misclassification of proteinuria status. Prior TB co-infection was also not significantly associated with rapid eGFR loss though some studies have found a high prevalence of CKD in PWH and TB (67).

Taken together, our findings have implications for long-term health outcomes in older PWH. CKD and rapid eGFR decline are associated with a higher risk of CVD, incident kidney failure and mortality (68, 69). Moreover, in Uganda and SSA, there is limited access and affordability of kidney replacement therapy (4). Our study findings underscore the need for early CKD detection, risk stratification and individualized kidney function monitoring especially in PWH who using TDF based ART. Our findings also underscore the importance of advocating for availability and selection of ART that prioritizes kidney safety among older PWH in Uganda and SSA.

Our study also has limitations. First, the follow-up was relatively short, precluding more significant observations in eGFR change. We also used creatinine-based eGFR, the accuracy of which may be affected by low muscle mass and limited physical activity in older PWH (70, 71) ; this may have underestimated the prevalence of CKD, biased the observed differences by sex, and distorted longitudinal patterns in eGFR. A study using cystatin C in this population along with creatinine is warranted to supplement the findings in this population.

In summary, RKFD is common and linked to cumulative TDF use among older PWH in SSA with minimal kidney function recovery. We recommend prioritizing more frequent or individualized kidney function monitoring and promoting availability and selection of ART that prioritizes kidney health for older PWH in SSA.

## Supplementary Material

Supplementary Files

This is a list of supplementary files associated with this preprint. Click to download.

• SupplementaryMaterialssubmittedbyAmutuhaireJudithSsemasaazi.pdf

• QuestionnareHASAcohort.pdf

## Figures and Tables

**Figure 1:**
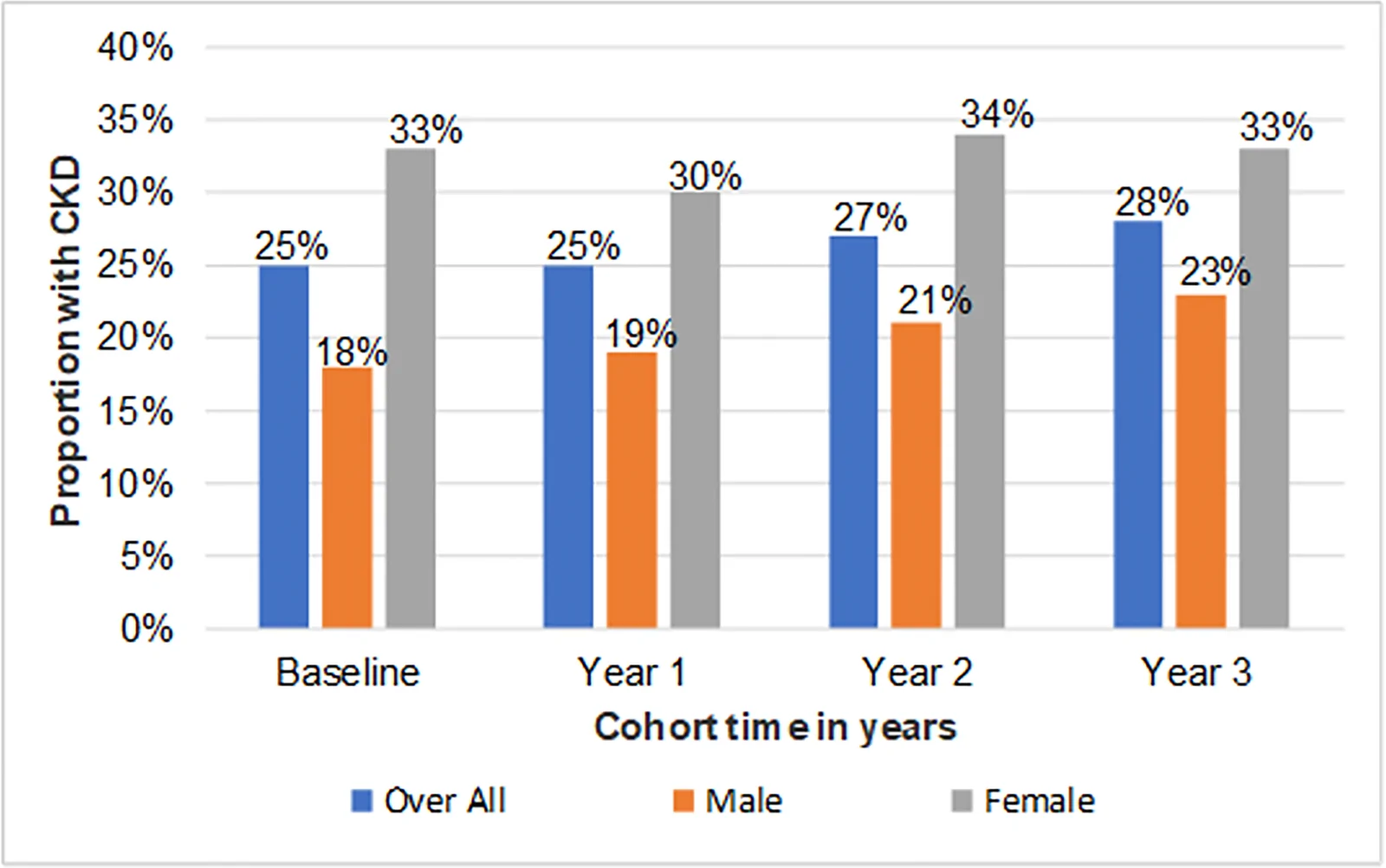
Prevalence of CKD among older PWH at baseline and during the cohort Predictors of changes in longitudinal eGFR

**Figure 2:**
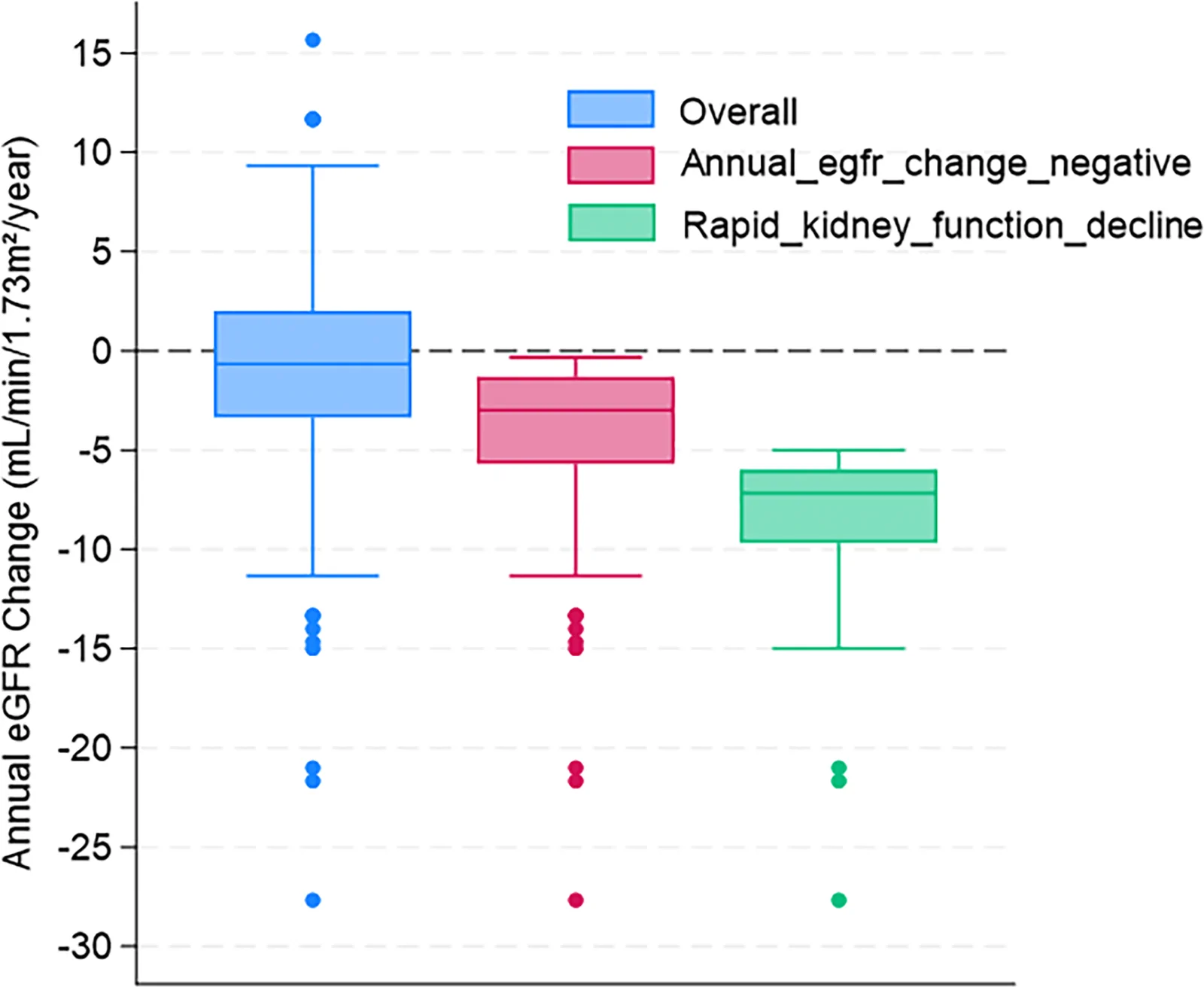
Annual eGFR change among older PWH stratified overall and by categories of eGFR decline

**Table 1:** Baseline characteristics of older PWH, stratified overall and by sex

Age in years, Median (IQR)	Overall, N (%) 500 (100%)	Males, n (%) 257 (51%)	Females, n (%) 243 (49%)	P-value
	64 (62, 68)	65 (62, 69)	64 (62, 68)	0.05*
eGFR in mls/min/1.73m^2^, Median (IQR)	75 (60,92)	80 (67,93)	70 (57,87)	**< 0.01** *
eGFR < 60mls/min/1.73m^2^	127 (25)	47 (18)	80 (33)	**< 0.01**
Systolic BP (mm Hg), Median (IQR)	133 (117,149)	138 (121,153)	128 (114,146)	**< 0.01** *
Diastolic BP (mm Hg), Median (IQR)	82 (74,90)	83 (74,91)	80 (73,90)	0.44*
BMI in kg/m^2^, Median (IQR)	24 (22, 28)	23 (21, 25)	26 (23, 30)	**< 0.01** *
NSAIDs use	24 (5)	11 (4)	13 (5)	0.58
At least one NCD	383 (77)	207 (81)	176 (72)	**0.03**
Hypertension	375 (75)	203 (80)	172 (71)	**0.03**
Diabetes mellitus	70 (14)	41 (16)	29 (12)	0.20
Stroke	8 (2)	4 (2)	4 (2)	0.94
Smoking (current and past)	119 (24)	106 (41)	13 (5)	**< 0.01**
Current alcohol use	140 (28)	89 (35)	51 (21)	**< 0.01**
Prior diagnosis of TB	184 (37)	110 (43)	74 (31)	**< 0.01**
HBV surface antigen positive	21 (4)	18 (7)	3 (1)	**< 0.01**
CD4 T-lymphocytes, (cells/ml), Median (IQR)	645 (461,850)	554 (424,733)	726 (538,951)	**< 0.01** *
Viral load ≥ 50 HIV RNA copies/ml	40 (8)	25 (10)	15 (6)	0.15
Years on ART, Median (IQR)	14 (9, 16)	14 (9, 16)	13 (9, 16)	0.16*
ART regimen at baseline:				
TDF-based regimen	379 (76)	192 (75)	187 (77)	0.56
Abacavir-based regimen	69 (14)	37 (15)	32(13)	0.77
Protease inhibitors	93 (19)	46 (18)	47 (19)	0.71
Integrase strand transfer inhibitors	327 (66)	180 (66)	157 (65)	0.68
Ever used TDF	481 (96)	247 (96)	244 (96)	0.91
TDF use duration (years), Median (IQR)	5.0 (3.7,8.9)	5.1 (3.8,8.8)	5.0 (3.7,8.9)	0.83*
Switched from TDF	174 (35)	77 (30)	97 (40)	**0.02**
Switched from TDF prior cohort enrollment	66 (14)	35 (14)	34 (14)	0.87
Switched from TDF during cohort	121 (24)	50 (19)	71 (29)	**0.01**
Proteinuria (≥30 mg/dl (+))	72 (15)	34 (13)	38 (16)	0.44

The p-values* are based on Wilcoxon rank sum test for continuous variables while all the other p-values are based on a chi square or Fishers Exact test for categorical variables: PWH, People with HIV; eGFR, estimated glomerular filtration rate; BP, Blood Pressure; TB, Tuberculosis; HBV, Hepatitis B virus; NCD, Non communicable disease; NSAIDS, Non-steroidal anti-inflammatory drugs; TDF, Tenofovir disoproxil fumarate; ART, Antiretroviral therapy; RNA, Ribonucleic acid; BMI, Body mass index.

**Table 2:** Predictors of annual eGFR change among older PWH

Characteristics of participants	Variables selected by BMA		Multivariable adjusted estimates (95%CI), p-value
	Unadjusted estimates (95%CI), p-value	Posterior probability	
Age (per decade)	0.22 (−0.77, 1.21), 0.66	2.6%	
Female	1.55 (0.66, 2.44), < 0.01	**94.2%**	1.82 (0.95, 2.70), **< 0.01**
Diastolic BP (per 10 mmHg)	0.04 (−0.30, 0.38), 0.81	1.5%	
Systolic BP (per 10 mmHg)	−0.12 (−0.33, 0.08), 0.24	3.2%	
BMI (per kg/m^2^)	−0.05 (−0.14, 0.05), 0.34	5.6%	
NSAIDS	1.28 (−0.36, 2.91), 0.13	2.3%	
At least one NCD	−0.46 (−1.35, 0.44), 0.32	3.6%	
Hypertension	−0.55 (−1.45, 0.35), 0.23	11.7%	
Diabetes mellitus	−1.43 (−3.07, 0.20), 0.09	4.4%	
Stroke	0.42 (−3.25, 4.09), 0.82	1.6%	
Smoking (past and current)	−0.29 (−1.30, 0.72), 0.57	4.8%	
Current alcohol use	−1.01 (−2.06, 0.04), 0.06	3.8%	
Prior diagnosis of TB	−0.51 (−1.43, 0.41), 0.28	1.6%	
HBV surface antigen positive	2.75 (0.34, 5.16), 0.03	**99.7%**	4.19 (2.11, 6.27), **< 0.01**
CD4 T-lymphocyte (per 100cells/ml higher)	0.23 (0.07, 0.38), < 0.01	28.6%	
Viral load ≥ 50 HIV RNA copies/ml	−1.10 (−2.36, 0.17), 0.09	2.7%	
Years on ART	−0.03 (−0.13, 0.07), 0.57	1.4%	
TDF-based regimen	0.44 (−0.79, 1.67), 0.49	2.4%	
Abacavir-based regimen	0.12 (−1.49, 1.74), 0.88	1.4%	
Protease inhibitors	−1.87 (−2.99, −0.75), < 0.01	1.9%	
Integrase strand transfer inhibitors	2.20 (1.23, 3.18), < 0.01	**100.0%**	2.50 (1.52, 3.48), **< 0.01**
Ever used TDF	−0.40 (−1.97, 1.17), 0.62	1.5%	
TDF switch	0.61 (−0.39, 1.61), 0.23	2.4%	
TDF duration in years	−0.02 (−0.14, 0.09), 0.67	2.0%	
TDF status at baseline:			
Current TDF use vs. never	1.43 (−0.88, 3.74), 0.23	2.1%	
Recent TDF use vs. never	0.14 (−2.79, 3.07), 0.92	9.2%	
Past TDF use vs. never	1.76 (−1.11, 4.63), 0.23	1.4%	
Dipstick Proteinuria	−0.10 (−0.49, 0.30), 0.63	2.2%	

Adjusted estimates are from a mixed effects linear regression model with random intercepts and slopes estimated following selection of variables by Bayesian model averaging (BMA). BMI (Body mass index) and viral load are time updated, all the other covariates are taken from baseline: PWH, People with HIV; eGFR, estimated glomerular filtration rate; BP, Blood Pressure; TB, Tuberculosis; HBV, Hepatitis B virus; NCD, Non-communicable disease; NSAIDS, Non-steroidal anti-inflammatory drugs; TDF, Tenofovir disoproxil fumarate; ART, Antiretroviral therapy; RNA, Ribonucleic acid.

**Table 3:** Risk factors for rapid kidney function decline among older PWH

Baseline characteristics	Rapid kidney function decline, n (%) 76 (15.2%)	Unadjusted RR (95% CI)	P-value	Adjusted RR (95%CI)	P-value
Age in years	65 (62–74)	1.02 (0.69–1.52)	0.90	0.98 (0.94–1.02)	0.38
Male gender	50 (66)	1.82 (1.17–2.83)	0.01	1.91 (1.08–3.37)	**0.03**
Diastolic BP (per 10mmHg)	81 (74–89)	0.97 (0.84–1.13)	0.73	0.89 (0.72–1.10)	0.29
Systolic BP (per 10mmHg)	137 (121–152)	1.05 (0.97–1.14)	0.23	1.10 (0.96–1.25)	0.18
BMI (kg/m^2^)	23.9 (21.6–28.2)	1.00 (0.96–1.04)	0.98	1.04 (0.99–1.09)	0.12
NSAIDS use	2 (3)	0.54 (0.14–2.06)	0.36	0.59 (0.21–1.68)	0.32
At least one NCD	62 (82)	1.00 (0.64–1.56)	0.99	0.80 (0.10–6.59)	0.84
Hypertension	61 (80)	1.36 (0.80–2.30)	0.26	1.36 (0.16–11.35)	0.78
Diabetes	13 (17)	1.27 (0.74–2.18)	0.39	0.94 (0.48–1.86)	0.86
Stroke	1 (1)	0.82 (0.13–5.20)	0.83	1.01 (0.25–4.06)	0.99
Smoking (past and current)	18 (24)	0.99 (0.61–1.61)	0.95	0.72 (0.42–1.20)	0.21
Current alcohol use	27 (36)	1.42 (0.92–2.17)	0.11	1.24 (0.80–1.92)	0.34
Prior diagnosis of TB	33 (44)	1.34 (0.88–2.03)	0.17	1.15 (0.72–1.85)	0.55
HBV surface antigen positive	2 (3)	0.62 (0.16–2.35)	0.48	0.29 (0.08–1.01)	0.05
CD4 T-lymphocytes (per 100cells/ml higher)	548 (376–729)	0.89 (0.82–0.97)	<0.01	0.91 (0.84–0.99)	**0.04**
Viral load ≥ 50 HIV RNA copies/ml	6 (8)	0.88 (0.43–1.79)	0.73	0.89 (0.48–1.68)	0.73
Years on ART	13 (9–16)	0.99 (0.94–1.04)	0.70	0.97 (0.92–1.02)	0.25
Abacavir based regimen	15 (20)	1.54 (0.93–2.55)	0.10	1.17 (0.51–2.65)	0.71
Protease inhibitor-based regimen	21 (28)	1.67 (1.07–2.62)	0.03	0.93 (0.51–1.69)	0.80
Integrase strand transfer inhibitor-based regimen	35 (46)	0.45 (0.30–0.68)	<0.01	0.41 (0.23–0.72)	**< 0.01**
TDF duration (log_2_TDF years)	5.5 (3.9–9.6)	1.17 (0.95–1.42)	0.14	1.33 (1.11–1.60)	**< 0.01**
Switched from TDF during cohort	17 (24)	0.92 (0.56–1.52)	0.74	1.14 (0.66–1.95)	0.64
Switched from TDF prior cohort enrollment	14 (19)	1.52 (0.90–2.56)	0.16	1.39 (0.56–3.41)	0.57
Proteinuria (≥30mg/dl (+))	14 (18)	1.34 (0.79–2.27)	0.27	1.23 (0.74–2.07)	0.43

Estimates are from a modified Poisson regression model: PWH, People with HIV; eGFR, estimated glomerular filtration rate; RR, Risk Ratio; BP, Blood Pressure; TB, Tuberculosis; HBV, Hepatitis B virus; NCD, Non communicable disease; NSAIDS, Non-steroidal anti-inflammatory drugs; TDF, Tenofovir disoproxil fumarate; ART, Antiretroviral therapy; RNA, Ribonucleic acid; BMI, Body mass index.

## Data Availability

Our manuscript is based on primary research data generated at the Infectious Diseases Institute and the data supporting these results can be available upon acceptance of the manuscript with reasonable requests made to the study team via the corresponding author.

## Abbreviations

ART: Antiretroviral therapy
BMI: Body mass index
CKD: Chronic kidney disease
CVD: Cardiovascular disease
eGFR: estimated glomerular filtration rate
ESKD: End stage kidney disease
FBG: Fasting blood glucose
HBV: Hepatitis B virus
HIV: Human immunodeficiency virus
HIVAN HIV: associated nephropathy
IDI: Infectious Diseases Institute
INSTIs: Integrase strand transfer inhibitors
KDIGO: Kidney disease improving global outcomes
LIC: Low income countries
PWH: People with HIV
RBG: Random blood glucose
RKFD: Rapid kidney function decline
RNA: Ribonucleic acid
SSA: Sub-Saharan Africa
TAF: Tenofovir alafenamide fumarate
TDF: Tenofovir disoproxil fumarate
UNAIDS: United Nations program on HIV/AIDS
